# Molecular Cloning, Tissue Distribution, and Pharmacological Characterization of GPR84 in Grass Carp (*Ctenopharyngodon Idella*)

**DOI:** 10.3390/ani13193001

**Published:** 2023-09-23

**Authors:** Yang Li, Wei-Jia Song, Shao-Kui Yi, Hui-Xia Yu, Hao-Lin Mo, Ming-Xing Yao, Ya-Xiong Tao, Li-Xin Wang

**Affiliations:** 1 College of Animal Science and Technology, Northwest A&F University, Yangling 712100, China; songvega@163.com (W.-J.S.); yuhuixia@nwsuaf.edu.cn (H.-X.Y.); 2017010832king@nwsuaf.edu.cn (H.-L.M.); ymx1114@nwsuaf.edu.cn (M.-X.Y.); fisherwanglx@nwsuaf.edu.cn (L.-X.W.); 2 College of Life Sciences, Huzhou University, Huzhou 313000, China; 02844@zjhu.edu.cn; 3 Department of Anatomy, Physiology and Pharmacology, College of Veterinary Medicine, Auburn University, Auburn, AL 36849, USA; taoyaxi@auburn.edu

**Keywords:** GPR84, medium-chain fatty acid, grass carp, expression, signaling

## Abstract

**Simple Summary:**

GPR84 is a G protein-coupled receptor expressed in immune cells. In mammals and amphibians, it is activated by medium-chain fatty acids and plays critical roles in inflammation and eye development. However, our understanding of GPR84 in fish remains limited. In this study, we successfully cloned the coding sequence of grass carp GPR84, revealing its high expression in the liver and spleen. In cells transfected with ciGPR84, we observed its responsiveness to medium-chain fatty acids such as capric acid, undecanoic acid, and lauric acid. Surprisingly, ciGPR84 did not respond to a synthetic activator called diindolylmethane. Notably, we found that lauric acid and capric acid exhibited the strongest activation and inhibition of ERK and cAMP signaling, respectively, suggesting their potential as immune modulators. These findings provide valuable insights for mitigating chronic inflammation in farmed fish, especially grass carp.

**Abstract:**

The G-protein-coupled receptor GPR84, activated by medium-chain fatty acids, primarily expressed in macrophages and microglia, is involved in inflammatory responses and retinal development in mammals and amphibians. However, our understanding of its structure, function, tissue expression, and signaling pathways in fish is limited. In this study, we cloned and characterized the coding sequence of GPR84 (ciGPR84) in grass carp. A phylogenetic analysis revealed its close relationship with bony fishes. High expression levels of GPR84 were observed in the liver and spleen. The transfection of HEK293T cells with ciGPR84 demonstrated its responsiveness to medium-chain fatty acids and diindolylmethane (DIM). Capric acid, undecanoic acid, and lauric acid activated ERK and inhibited cAMP signaling. Lauric acid showed the highest efficiency in activating the ERK pathway, while capric acid was the most effective in inhibiting cAMP signaling. Notably, DIM did not activate GPR84 in grass carp, unlike in mammals. These findings provide valuable insights for mitigating chronic inflammation in grass carp farming and warrant further exploration of the role of medium-chain fatty acids in inflammation regulation in this species.

## 1. Introduction

Fatty acids were traditionally viewed as metabolic intermediaries that primarily serve as an energy source. However, mounting evidence in recent years has unveiled their pivotal role as endogenous signaling molecules, orchestrating a wide range of physiological processes. A growing body of literature demonstrates that fatty acids possess the ability to regulate insulin secretion, impact cellular apoptosis, and modulate inflammatory and immune responses [1,2]. Free fatty acids include short-chain fatty acids (SCFAs) (C1–C8), medium-chain fatty acids (MCFAs) (C9–C14), and long-chain fatty acids (LCFAs) (more than C14). SCFAs in animals, primarily produced by intestinal microorganisms fermenting cellulose, improve inflammatory diseases and allergic asthma, and promote the maturation of T helper cells [3,4]. MCFAs, ingested directly from food [5], have been shown to stimulate the differentiation of Th1 and Th17 cells, thereby promoting an inflammatory response [6]. LCFAs, derived from milk and aquatic products, inhibit macrophage-induced tissue inflammation, with anti-inflammatory activity [3,7].

Fatty acids primarily exert the aforementioned physiological functions through a class of cell membrane-bound G protein-coupled receptors (GPCRs), known as free fatty acid receptors (FFARs). Upon sensing fatty acid signals, FFARs activate the second messenger system, thereby triggering physiological regulatory effects [1]. Nature has developed distinct FFARs to bind fatty acids with different carbon chain lengths. For example, GPR43 is a SCFA receptor that can affect T-helper cell function [8] and leukocyte chemotaxis [9]. GPR120 is a LCFA receptor that participates in the regulation of obesity-related inflammation after being activated by omega-3 fatty acids [10]. The GPR84 receptor is an MCFA receptor promoting inflammation [11]. A recent study showed that GPR84 is highly expressed in the inflamed colon tissues of active ulcerative colitis (UC) patients and dextran sulfate sodium (DSS)-induced colitis mice, and that the knockout of GPR84 could help mice resist the development of colitis induced by DSS [12]. In addition, under non-pathological conditions, GPR84 is highly expressed in activated mouse macrophages [13] and is significantly increased in response to lipopolysaccharide (LPS) stimulation [14]. Th2 cytokines, such as IL-4 with anti-inflammatory activity, are increased in T cells from GPR84 knockout mice [15].

The pharmacology of GPCRs is a widely researched topic due to their ubiquitous presence and importance as drug targets [16]. This field of study aims to investigate the interactions and effects of ligands or drugs with GPCRs, encompassing a comprehensive understanding of the activation and signal transduction mechanisms of GPCRs, as well as the binding mechanisms of ligands to GPCRs, and the modulation of their activities [17]. Pharmacological investigations of fish GPCRs were initiated by the pioneering work of Richard Peter, as reported in Cerdá-Reverter et al. [18]. Peter and his colleagues transfected the goldfish melanocortin-4 receptor (MC4R) into human embryonic kidney 293 (HEK293) cells, and used radiolabeled [Nle4, D-Phe7]-α-MSH (NDP-MSH), a synthetic potent agonist of MC4R, to probe the binding activity of goldfish MC4R and its downstream effect on cAMP concentration. Subsequently, other researchers employed HEK-293 cells and Chinese hamster ovary (CHO) cells to investigate the pharmacology of diverse fish GPCRs, including melanocortin receptors in species such as the channel catfish (*Ictalurus punctatus*) [19], spotted scat (*Scatophagus argus*) [20], grass carp (*Ctenopharyngodon idella*) [21], snakehead (*Channa argus*) [22], ricefield eel (*Monopterus albus*) [23], topmouth culter (*Culter alburnus*) [24], rainbow trout (*Oncorhynchus mykiss*) [25], and elephant shark (*Callorhynchus milii*) [26]. These studies, conducted in mammalian cells as a platform, not only elucidated the functional regulation of fish GPCRs on downstream signaling, but also revealed distinct characteristics of fish GPCRs, such as higher constitutive activity compared to their human orthologs [27].

In addition to MCFAs, diindolylmethane (DIM) [28], embelin [29], and 6-N-octylaminouracil (6-OAU), are all alternative agonists of GPR84, and 6-OAU is the most potent agonist [14]. 6-OAU rapidly induces microglia cell folding and increases microglia cell movement by stimulating mouse GPR84 [30]. The inflammatory mediator CXCL1 is increased in rats injected with 6-OAU [31]. These experiments further demonstrated that the function of GPR84 is associated with inflammation and macrophage activity. Human and mouse GPR84 is coupled to Gi/o protein, with receptor activation resulting in decreased intracellular cAMP accumulation, which is inhibited by pertussis toxin [11]. When stimulated by 6-OAU, GPR84 also increases the phosphorylation of Akt and ERK and the nuclear translocation of NF-κB in macrophages and promotes inflammation by increasing the expression of key cytokines and chemokines [14].

Compared with studies in mammals, there are only three reports on fish GPR84. Huang et al. [32] reported that zebrafish GPR84 is involved in the accumulation of lipid droplets and can up-regulate the expression of IL-12 p40 upon undecanoic acid treatment. The authors of [33] showed that the overexpression of zebrafish GPR84 in the RAW264.7 cell line can enhance phagocytic activity. Another study found that the brain of rainbow trout can sense MCFAs such as octanoate through GPR84, which can subsequently affect their food intake [34]. These findings suggest that, akin to mammals, GPR84 plays a role in regulating the immune system of fish and may also be partially involved in maintaining energy homeostasis.

GPR84 plays a crucial role in regulating the immune system of fish, making it an attractive target for drug development and physiological regulation to mitigate the incidence of metabolic diseases in farmed fish. Among the fish species affected by metabolic inflammation, the grass carp stands out as a significant example. The grass carp holds great economic importance in China, boasting the highest yield globally [35]. However, due to high-density culture practices and the widespread use of compound feed, cultured grass carp often experience chronic inflammation accompanied by various metabolic disorders. Consequently, investigating the potential involvement of MCFAs in regulating chronic inflammation in grass carp becomes an area of significant interest. In this study, we successfully cloned the full-length coding region of grass carp GPR84, determined its tissue expression pattern, and demonstrated its capacity to modulate downstream signaling pathways upon MCFA stimulation.

## 2. Materials and Methods

### 2.1. Chemicals, Reagents, and Plasmids

To elucidate the pharmacological properties of grass carp GPR84 (ciGPR84), we employed DIM as a representative agonist, known for its potent activation of GPR84 in mammalian systems. Additionally, considering their potential endogenous role, we investigated the activation potency of three commonly occurring MCFAs in gastrointestinal tracts: capric acid, undecanoic acid, and lauric acid. These chemicals were obtained commercially from Shanghai Macklin Biochemical Technology (Shanghai, China), and their purities are as follows: DIM (purity ≥ 98%), capric acid (purity ≥ 99.5%), undecanoic acid (purity ≥ 99%), and lauric acid (purity ≥ 99.5%).

Since the aforementioned compounds are insoluble in water, they were initially dissolved in 3% dimethyl sulfoxide (DMSO), then coated with 2% bovine serum albumin (BSA) to create a stock solution with a concentration of 10 mM. Prior to use, the stock solution was diluted to the working concentration using a serum-free DMEM medium. The concentration of fatty acids in the solution was detected using a Tecan F200 microplate reader (Tecan, Männedorf, Switzerland) through colorimetric analysis. The luciferase assay kits were obtained from Beyotime Biotechnology (Shanghai, China). The plasmids pGL4.29 and pGL4.33 used for examining signaling were obtained from Promega (Madison, WI, USA).

### 2.2. Total RNA Extraction and cDNA Synthesis

Grass carp specimens were obtained from Western Orchid Ecological Park (Yangling, China). A total of 9 grass carp individuals, with an average body length (SD) of 35.2 ± 4 cm and an average weight (SD) of 674.4 ± 57 g, were included in this study. Prior to sacrifice, the grass carp were temporarily reared for three days. Due to the immaturity of the grass carp used in this study, it was not feasible to differentiate their sexes based on their gonads. The fish were anesthetized using a 1:500 dilution of 2-phenoxyethanol, followed by prompt euthanasia and subsequent dissection. Tissues including kidney, brain, heart, gill, liver, spleen, intestines, and muscle were collected for RNA extraction. For the RNA extraction, tissues of the same type from different individuals were combined to create a pooled sample. Total RNA was extracted using the RNA pure Tissue & Cell Kit (CWBIO, Shanghai, China). The quality of the RNA extraction was assessed using a NanoDrop ND-1000 spectrophotometer (NanoDrop Technologies, Wilmington, DE, USA), and a minimum OD260/280 ratio of 1.9 was considered satisfactory for quality assurance. To remove any DNA contamination, a Turbo DNase kit (Ambion, Austin, TX, USA) was utilized, and the resulting RNA was employed for cDNA synthesis.

The RNA was reverse transcribed into cDNA using the Revert Aid RT Reverse Transcription Kit (Thermo Fisher Scientific, Waltham, MA, USA). For the reverse transcription process, a premix containing 5 µg of total RNA and 1 µL of oligo-deoxythymidine was prepared in a total volume of 12 µL. The mixture was incubated at 70 °C for 4 min and immediately cooled on ice for 2 min. Subsequently, 5× reaction buffer, 20 mM deoxynucleotide triphosphate (dNTP), and 200 U of Moloney murine leukemia virus reverse transcriptase (M-MLV) were added to the mixture, bringing the final reaction volume to 20 µL. The reverse transcription reaction was carried out at 42 °C for 1 h, followed by heating at 70 °C for 5 min to inactivate the reaction. The resulting cDNA products were stored at −20 °C and used for subsequent molecular cloning and tissue-specific quantitative expression studies of ciGPR84.

### 2.3. Molecular Cloning of Grass Carp GPR84

The predicted nucleotide sequence of grass carp *gpr84* used for designing primers was acquired from the Grass Carp Genome Database [36]. The PCR procedure was performed at the following conditions: 95 °C for 5 min, followed by 32 cycles at 95 °C for 30 s, 60 °C for 30 s, and 72 °C for 45 s, as well as a final 10 min extension at 72 °C. The primer sequences are shown in Table 1 and were synthesized by AUGCT (Beijing, China). The PCR product containing the entire open reading frame (ORF) was subcloned into the pcDNA3.1(+) (pciGPR84) and used for transfection.

### 2.4. Homology, Phylogenetic, and Chromosome Synteny Analysis of ciGPR84

Multiple alignments of amino acid sequences of GPR84s from different species were performed with the ClustalW multiple sequence alignment program by MEGA X software. The putative disulfide bridges of ciGPR84 were predicted by PROSITE (https://prosite.expasy.org/, accessed on 22 April 2022). The putative transmembrane domains (TMDs) of ciGPR84 were predicted by the Conserved Domain Search Service (https://www.ncbi.nlm.nih.gov/Structure/cdd/wrpsb.cgi, accessed on 22 April 2022). A phylogenetic tree was constructed using the neighbor-joining (NJ) method in MEGA X software (version 10.2), based on alignments of amino acid sequences of GPR84. Bootstrap values were estimated from 1000 replicates to assess the robustness of the tree. A chromosome synteny analysis was performed between several fish and mammalian species with the Genomicus (http://www.genomicus.biologie.ens.fr/genomicus, accessed on 22 April 2022) and National Center for Biotechnology Information (NCBI) genome browser (https://www.ncbi.nlm.nih.gov, accessed on 22 April 2022).

### 2.5. Real-Time PCR for Tissue Expression

The expression of grass carp *gpr84* mRNA in various tissues was assessed using a real-time PCR (RT-PCR) technique. The primers for the RT-PCR procedure, synthesized by AUGCT (Beijing, China), are listed in Table 1. The PCR cycling conditions included an initial denaturation at 95 °C for 5 min, followed by 40 cycles of denaturation at 95 °C for 10 s and annealing/extension at 60 °C for 30 s. A dissociation curve analysis was performed to confirm the specificity of the amplified products. To normalize the mRNA expression across different tissues, the housekeeping gene *actb* (encoding β-actin) was used as an internal control. The relative expression levels of grass carp GPR84 were calculated using the comparative threshold cycle method (2^−ΔΔCt^) [37].

### 2.6. Cell Culture

Pharmacological studies of GPCRs generally utilize mammalian cells to avoid the presence of endogenous GPCRs and achieve higher expression efficiency on the cell membrane. HEK293 and its derivative cell lines, such as HEK293T, as well as CHO cells, are the most commonly used cell lines in this field of research [19,22,26,38]. In this study, we employed HEK293T cells as the research platform according to a method we established previously [39].

Human embryonic kidney cells HEK293T obtained from Beyotime Biotechnology (Shanghai, China) were cultured with Dulbecco’s Modified Eagle Medium (DMEM) containing a 10% fetal bovine serum in an incubator (37 °C and 5% CO_2_ humidified atmosphere). The cells were plated into 24-well plates and cultured for 24 h before assays. The Countess 3 cell counter (Invitrogen, Waltham, MA, USA) was used for cell counting.

### 2.7. Functional Characterization of ciGPR84 in Cultured HEK293 Cells

The functionality of ciGPR84 was assessed using our established method [40]. Specifically, the coding sequence (CDS) of ciGPR84 was subcloned into the pcDNA3.1 (+) vector (Invitrogen, Carlsbad, CA, USA), resulting in the construction of pcDNA3.1-ciGPR84 (pciGPR84). HEK293T cells were co-transfected with pciGPR84 (or pcDNA3.1 empty vector), two luciferase reporter vectors (pGL4.29 and pGL4.33) containing a cAMP response element (CRE) and serum response element (SRE), respectively, and pEGFP-N1 (as an internal control for transfection normalization) in a mixture with polyethyleneimine (PEI) transfection reagent (Fusheng Biotechnology, Shanghai, China). The cells were then incubated in the original medium for 24 h, transferred to a 48-well plate, and grown for an additional 24 h to reach a cell density of 2 × 10^5^ cells per well. For cells transfected with pGL4.29 and pGL4.33, four ligands (DIM, capric acid, undecanoic acid, and lauric acid) were diluted to working concentrations in a serum-free DMEM medium and added to the 48-well plates to treat the cells for 6 h. It is important to note that due to the anticipated inhibitory effect of MCFA on the intracellular cAMP, 5 μM forskolin was added to all wells transfected with pGL4.29 to pre-activate the cellular adenylyl cyclase.

After the treatment, cells were lysed with 1 × passive lysis buffer (Beyotime Biotechnology, Shanghai, China), and a luciferase substrate was added to initiate a glow-generating reaction. The luminescence signals were measured using a Tecan F200 (Tecan, Männedorf, Switzerland) microplate reader to determine the relative luciferase activity in each well. Three independent experiments were performed for each agonist concentration (*n* = 3), and the data were presented as a mean ± SEM.

### 2.8. Statistical Analysis

According to our previously established method [40], the luciferase activities were converted to fold change relative to the control group (DMEM serum-free medium) and fitted to a dose-response curve using a nonlinear regression analysis. A data analysis was performed using GraphPad Prism 7 software (GraphPad Software, San Diego, CA, USA).

## 3. Results

### 3.1. Analysis of the Full Length of the Sequence

**Figure 1:**
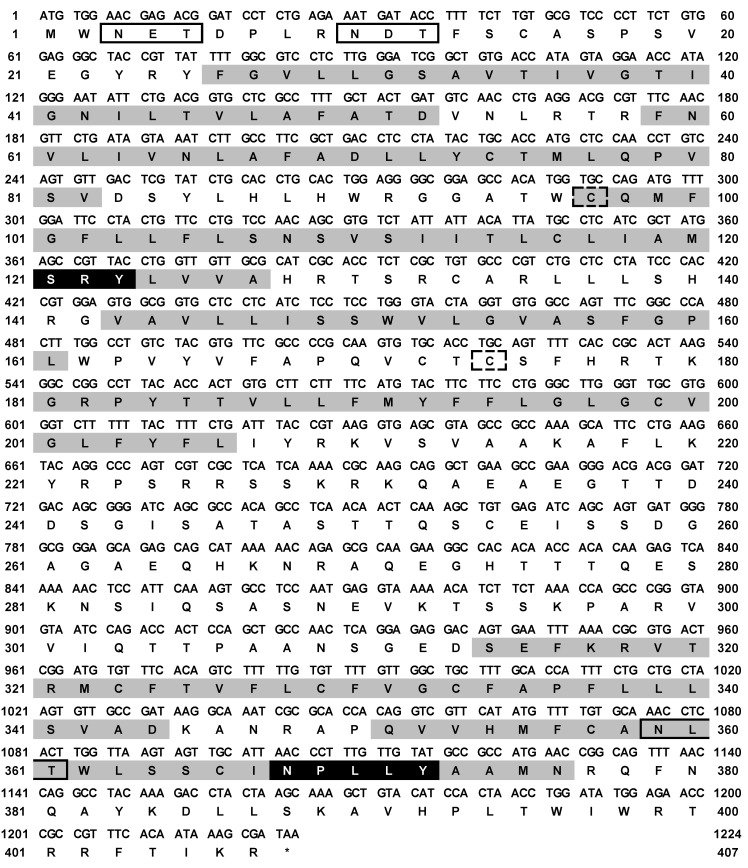
Nucleotide and deduced amino acid sequence of ciGPR84. Positions of nucleotide and amino acid sequences are marked on both sides. The seven TMDs are shaded in grey. The conserved motifs, SRY and NPxxY, are highlighted in black. Open boxes frame the presumptive *N*-glycosylation sites, and the boxes outlined by dash lines indicate the cysteines that possibly form disulfide bonds. Asterisk (*) denotes stop codon.

**Figure 2:**
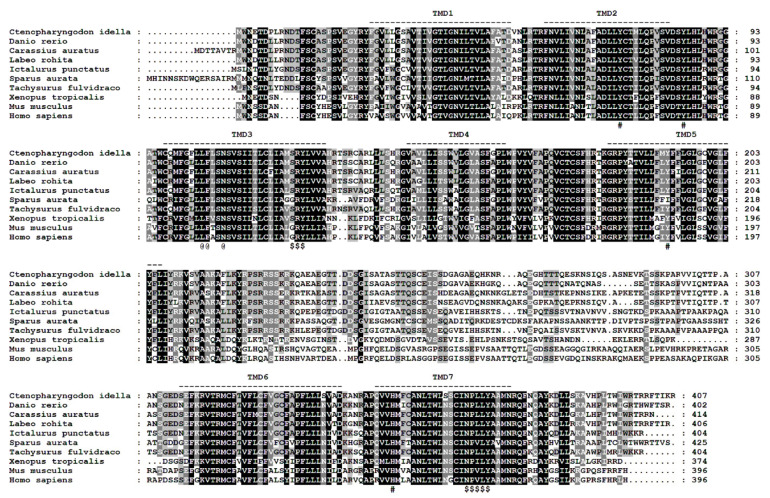
Multiple amino acid sequence alignments of GPR84s. Dashed lines represent the seven TMDs. The black shading represents amino acid sequences with 100% identity, while the grey shading indicates sequences with identity greater than 60% in the amino acid sequence alignment. @ indicates the amino acid residues essential for decanoic acid binding. # denotes amino acid residues that are capable of hydrogen bonding and may participate in the formation of the ligand-binding pockets. $ represents the conserved SRY and NPxxY motifs. GenBank accession numbers of the GPR84s: *Ctenopharyngodon idella* (MW535309), *Danio rerio* (NP_001099167.1), *Carassius auratus* (XP_026115535.1), *Labeo rohita* (RXN07797.1), *Ictalurus punctatus* (XP_017336000.1), *Sparus aurata* (XP_030274675.1), *Tachysurus fulvidraco* (XP_027029919.1), *Xenopus tropicalis* (NP_001072335.1), *Mus musculus* (NP_109645.1), *Homo sapiens* (NP_065103.1).

The ciGPR84 sequence contains an ORF of 1224 bp encoding 407 amino acids (GenBank Accession No. MW535309), and the molecular weight is 45.37 kDa. The structure of ciGPR84 has seven typical TMDs. The *N*-glycosylation site prediction shows that ciGPR84 has three Asn sites at 3, 10, and 359. A3 and A10 are outside the plasma membrane, which are highly likely glycosylation sites (Figure 1). The results of the disulfide bond prediction show that a disulfide bond could be formed between C97 and C174. The TMD3 and TMD7 of ciGPR84 contain a conserved SRY motif and NPxxY motif, respectively. The results of the multi-sequence alignment showed that, compared with mammals, fish GPR84 was relatively conserved in the TMDs and the extracellular loops, but showed low similarity in the intracellular loops. We found that three amino acids essential for decanoic acid binding in human GPR84 (L100, F101, and N104) are also conserved in ciGPR84 (L104, F105, and N108). Similarly, other residues (Y69, Y81, Y186, H352) that have hydrogen bonding capability in human GPR84 and may participate in the formation of ligand-binding pockets can also be found in ciGPR84 (Y73, Y85, Y192, and H354) (Figure 2).

### 3.2. Phylogenetic and Synteny Analysis of ciGPR84

**Figure 3:**
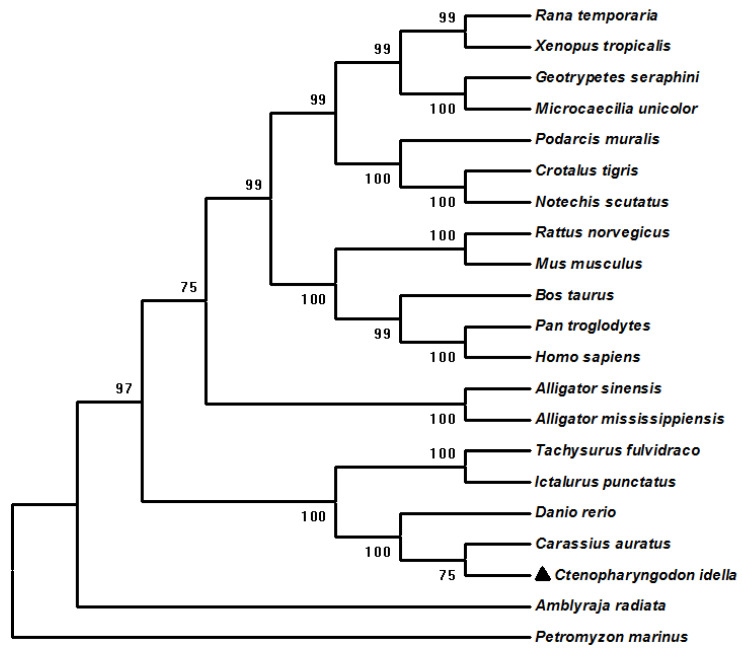
Phylogenetic tree of GPR84s. The tree was constructed by using the neighbor-joining (NJ) method. Numbers at nodes indicate the bootstrap value, as percentages, obtained for 1000 replicates. *Petromyzon marinus* was set as the outgroup. The black triangle indicates ciGPR84. GenBank accession numbers of the GPR84s: *Rana temporaria* (XP_040196684.1), *Xenopus tropicalis* (NP_001072335.1), *Geotrypetes seraphini* (XP_033791500.1), *Microcaecilia unicolor* (XP_030051340.1), *Podarcis muralis* (XP_028565013.1), *Crotalus tigris* (XP_039186530.1), *Notechis scutatus* (XP_026533155.1), *Rattus norvegicus* (XP_006242511.1), *Mus musculus* (NP_109645.1), *Bos taurus* (NP_001033657.1), *Pan troglodytes* (XP_522412.1), *Homo sapiens* (NP_065103.1), *Alligator sinensis* (XP_025047282.1), *Tachysurus fulvidraco* (XP_027029919.1), *Ictalurus punctatus* (XP_017336000.1), *Danio rerio* (NP_001099167.1), *Carassius auratus* (XP_026115535.1), *Ctenopharyngodon idella* (MW535309), *Amblyraja radiate* (XP_032872727.1), *Petromyzon marinus* (XP_032801560.1).

**Figure 4:**
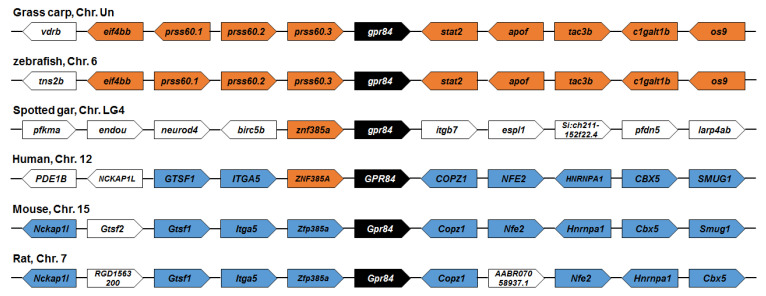
Synteny analyses of GPR84s. The syntenic genes are displayed as boxes with directions and linked by lines. GPR84 genes are shown in black boxes. The genes exhibiting conserved synteny in fishes are indicated in orange boxes, and those in mammals are shown in blue boxes.

We performed a phylogenetic analysis of ciGPR84 and other GPR84s. Grass carp GPR84 was more closely related to those of rohu, crucian carp, and zebrafish, than to those of amphibians and mammals (Figure 3). A synteny analysis showed that the adjacent genes, *os9* and *apof*, in grass carp were also found in rainbow trout. However, no conserved adjacent genes were observed between grass carp and human and rat GPR84s (Figure 4).

### 3.3. The Expression of Grass Carp GPR84 in Grass Carp

**Figure 5:**
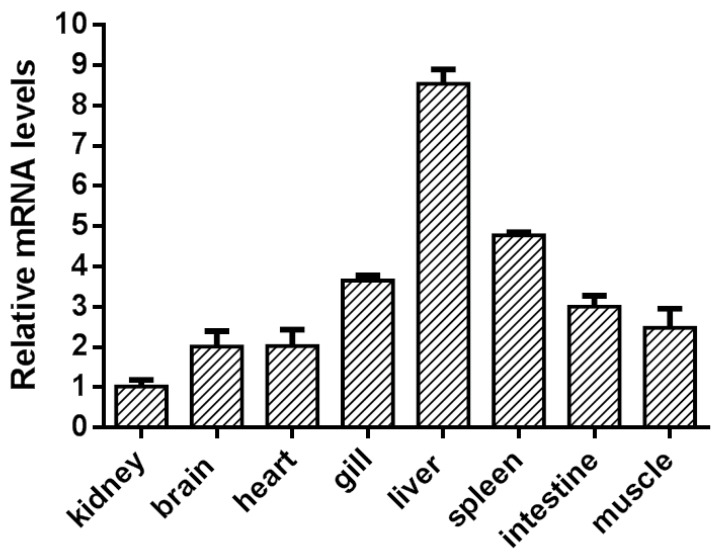
Tissue expression of grass carp GPR84 in different tissues. Quantitative real-time PCR analysis of GPR84 and *actb* mRNA levels in grass carp was performed. *actb* served as an internal reference gene to normalize the mRNA expression in different tissues. Fold difference was calculated by the 2^−ΔΔCt^ method. All the other tissues were compared with the kidney. Vertical bars represent the mean ± SD (*n* = 3).

The tissue expression of grass carp *GPR84* was determined by a real-time PCR procedure. Grass carp *gpr84* mRNA had the highest expression in the liver, followed by the spleen. The expression in other tissues, including the brain, gill, heart, intestine, kidney, and muscle, was lower than that of the liver and spleen (Figure 5).

### 3.4. Functional Characterization of ciGPR84 in Cultured HEK293 Cells

**Figure 6:**
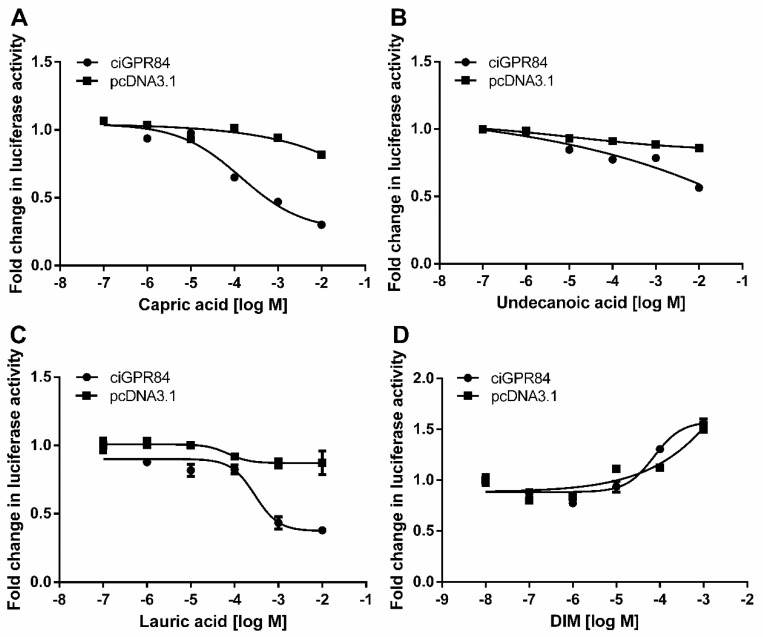
Activation of cAMP signaling mediated by ciGPR84. Induction of the luciferase activities of HEK293T cells expressing ciGPR84 in responses to different concentrations of MCFAs and DIM treatment. The cells were co-transfected with pGL4.29 and p ciGPR84 for 24 h and treated with agonists for 6 h. (**A**) capric acid (10^−7^ to 10^−2^), (**B**) undecanoic acid (10^−7^ to 10^−2^), (**C**) lauric acid (10^−7^ to 10^−2^), (**D**) DIM (10^−8^ to 10^−3^) treatments are shown. The co-transfected empty pcDNA3.1 and pGL4.29 were used as a control group. Each data point represents the mean ± SEM of 3 independent experiments.

We co-transfected HEK293 cells with pciGPR84 and pGL4.29 or pGL4.33, and treated the cells with capric acid, undecanoic acid, lauric acid, or DIM to investigate whether the different agonists could activate ciGPR84. As shown in Figure 6, the treatment of MCFAs could inhibit forskolin-induced cAMP levels in a dose-dependent manner in cells co-transfected with pciGPR84 and pGL4.29. The inhibitory potency of MCFAs on cAMP through ciGPR84 was quantified using the IC_50_ (half-maximal inhibitory concentration) value. The potency order of the three fatty acids was capric acid (IC_50_: 97.9 µM) > lauric acid (IC_50_: 301 µM) > undecanoic acid (IC_50_: 1490 µM). The DIM treatment did not lead to the inhibition of cAMP generation.

**Figure 7:**
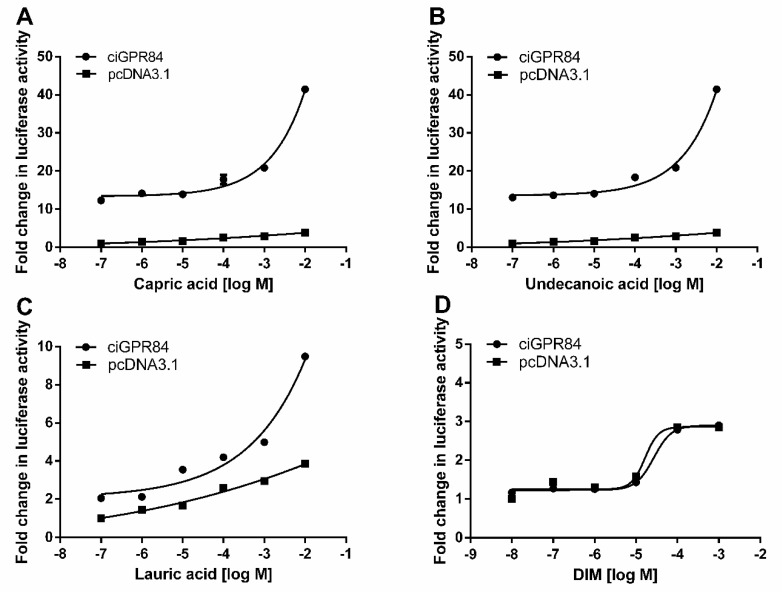
Activation of ERK signaling mediated by ciGPR84. Induction of the luciferase activities of HEK293T cells expressing ciGpr84 in responses to different concentrations of MCFAs and DIM treatment. HEK293T cells were co-transfected with pGL4.33 and ciGPR84 vectors for 24 h and treated with agonists for 6 h. Shown are (**A**) capric acid (10^−7^ to 10^−2^), (**B**) undecanoic acid (10^−7^ to 10^−2^), (**C**) lauric acid (10^−7^ to 10^−2^), (**D**) DIM (10^−8^ to 10^−3^). Cells co-transfected with empty vectors, pcDNA3.1 and pGL4.33, were used as the control group. Each data point represents the mean ± SEM of 3 independent experiments.

We used the pGL4.33 (pGL4-SRE-luciferase) reporter system to detect the activation of three MCFAs and DIM on the ERK signaling of cells transfected with ciGPR84. The results showed that all three MCFAs could activate this signaling pathway in a dose-dependent manner. The potency order of the three fatty acids was lauric acid (EC_50_: 4250 µM) > capric acid (EC_50_: 13,000 µM) > undecanoic acid (EC_50_: 13,800 µM). DIM did not activate the ERK signaling (Figure 7).

## 4. Discussion

In this study, we cloned the full-length CDS region of grass carp GPR84, analyzed the nucleotide and amino acid sequences, determined its tissue expression pattern, and confirmed downstream signaling pathways regulated by ciGPR84 using the luciferase reporter system.

Grass carp GPR84 cDNA was 1224 bp long and encoded a protein of 407 amino acids, similar to that of fish such as rohu (406 amino acids) and zebrafish (402 amino acids), and different from those of mammals (396 amino acids for human and mouse GPR84s). The multi-sequence alignment of GPR84s of different species showed that ciGPR84 was highly conserved in the seven TMDs and three extracellular loops. Grass carp GPR84 had a higher homology with those of crucian carp, South Asian scaly mackerel, and zebrafish, but a lower homology with those of mammals. The same conclusion was reached in the evolutionary tree constructed by the NJ method. These results collectively demonstrate the evolutionary conservation of GPR84 in bony fish.

Despite the relatively lower homology between ciGPR84 and human GPR84, key motifs and amino acids identified in human GPR84 are also present in ciGPR84. For instance, the highly conserved NPxxY motif in TMD7 is found in ciGPR84, and the tyrosine within this motif potentially interacts with the tyrosine at position 7.53 of the activated receptor, suggesting a typical activation mechanism observed in rhodopsin-family receptors [28]. Moreover, certain crucial amino acids (such as L104, F105, and N108) in ciGPR84, known to impact decanoic acid binding and hydrogen bond formation in human GPR84 [28], indicate the evolutionary conservation of the receptor’s role in fatty acid binding and receptor function. Additionally, the SRY motif, which appears as SRY in fish and amphibian GPR84 but as DRY in mammals, is observed in ciGPR84, exhibiting consistency with other fish species and suggesting motif conservation in lower vertebrates.

Studies have shown that GPR84 has the highest expression in macrophages and peripheral white blood cells and is also highly expressed in the mouse spleen [11]. In zebrafish, GPR84 is highly expressed in the intestinal tract and liver, which are also involved in immune defense in zebrafish [32]. In grass carp, the expression of GPR84 in the liver and spleen was significantly higher than that in other tissues, similar to the distribution of GPR84 in mice. Both the liver and spleen are essential immune organs in fish and mice, suggesting that ciGPR84 may be involved in the regulation of inflammation, similar to mammals. 

GPR84 can enhance phagocytic activity and promote an inflammatory response through Gαi protein [41]. We showed that MCFAs could activate the Gαi-cAMP and ERK signaling pathways through ciGPR84. The results showed that capric acid activated the Gαi-cAMP signaling pathway through ciGPR84 better than the other ligands, whereas lauric acid activated the ERK signaling pathway better than the other ligands. When compared to mouse GPR84, which exhibited EC50 values of 52, 4, 8, and 9 µM for nonanoic acid, capric acid, undecanoic acid, and lauric acid, respectively [11], ciGPR84 demanded higher ligand concentrations for activation. Based on these findings, although the precise levels of various MCFAs in the intestines of grass carp under high-density culture conditions remain unclear, reducing the content of capric acid from dietary sources may serve as an effective strategy to mitigate inflammatory responses in grass carp farming.

In mammals, the small molecule DIM acts as an agonist for GPR84 [42], but our results showed that DIM could not activate ciGPR84, likely due to the structural differences between ciGPR84 and mammalian GPR84s. We observed a similar phenomenon with fish melanocortin-4 receptors (MC4Rs), where peptide ligands act similarly but small molecules act differently in fish and mammalian MC4Rs. For example, THIQ (*N*-[(3*R*)-1,2,3,4-tetrahydroisoquinolinium3-ylcarbonyl]-(1*R*)-1-(4-chlorobenzyl)-2-[4-cyclohexyl-4-(1*H*-1,2,4-triazol-1-ylmethyl)piperidin-1-yl]-2-oxoethylamine), is an orthosteric agonist of human MC4R but allosteric agonist of spotted scat MC4R [20], ricefield eel MC4R [43], and spotted sea bass MC4R [44]. By comparing the effects of four human MC4R antagonists, including endogenous peptide antagonists, agouti-related peptides, and synthetic small molecule antagonists, on the cAMP and ERK1/2 signaling of two fish MC4Rs, spotted scat MC4R and grass carp MC4R, we showed that AgRP acts as an inverse agonist in cAMP signaling pathway in both fish MC4Rs, similar to human MC4R; but the small molecule ligands have effects that are different from human MC4R, with some even different between the two fish MC4Rs [27]. These results suggest that either in research or production application, ligands developed in mammalian systems need to be tested in the aquaculture species of interest. Extrapolation from mammalian system to fish system is not feasible.

In conclusion, we cloned ciGPR84, and analyzed its tissue expression pattern and signaling properties. Grass carp GPR84 was highly expressed in the liver and spleen. Functional studies demonstrated that decanoic acid, undecanoic acid, and lauric acid could inhibit cAMP generation and activate ERK signaling. However, DIM, the highly potent agonist of mammalian GPR84, could not activate ciGPR84. The findings of this study provide insights into the regulation of inflammatory responses in farmed grass carp through nutrient modulation and lay the foundation for further research on the role of MCFAs in chronic inflammation in fish.

## Figures and Tables

**Table 1:** Primers used for cloning and RT-PCR.

Primer Name	Primer Sequence (5′-3′)	Product Size (bp)	Ta ^1^ (°C)
ciGPR84-F	ATGTGGAACGAGACGGA	1224	53
ciGPR84-R	AGCTTTAGGTCATATTCGGA		
qPCR-F	CTGATAGTAAATCTTGCCTTCGC	194	60
qPCR-R	ACAACCAGGTAACGGCTCATAG		
ciβ-actin F	CGTGACATCAAGGAGAAG	215	57
ciβ-actin R	GAGTTGAAGGTGGTCTCAT		

^1^ Ta, annealing temperature.

## Data Availability

Data will be made available on request.
